# Challenges in Diagnosis of Lepidic Subtype in Lung Cancer According to W.H.O Classification: A Case Report

**DOI:** 10.3389/bjbs.2026.16119

**Published:** 2026-04-02

**Authors:** Cuong Pham Nguyen, Long Thien Phan, Phuong Thi Phan, Do Quyen Thi Phan, Tuong Pham Nguyen, Thoi Van Dang

**Affiliations:** 1 Department of Pathology, Hue Central Hospital, Hue, Vietnam; 2 Department of Oncology, International Medical Center, Hue Central Hospital, Hue, Vietnam; 3 Pulmonary Disease Department, Hue Central Hospital, Hue, Vietnam; 4 Oncology Center, Hue Central Hospital, Hue, Vietnam; 5 Da Nang University of Medical Technology and Pharmacy, Da Nang, Vietnam

**Keywords:** EGFR mutations, lepidic adenocarcinoma, osimertinib, pathology, respiratory Insufficiency

## Abstract

**Background:**

Lepidic adenocarcinoma of the lung often presents with atypical radiological patterns mimicking pneumonia, posing significant diagnostic challenges, particularly from small cytology samples. This can lead to delayed diagnosis and advanced-stage presentation.

**Case presentation:**

A 57-year-old non-smoking female presented with a persistent cough, massive sputum production, and diffuse bilateral pulmonary lesions on computed tomography scan, resembling pneumonia. The patient’s condition rapidly deteriorated into acute respiratory failure unresponsive to antibiotics. A sputum cell block confirmed lung adenocarcinoma (CK7+, TTF-1+). Liquid biopsy via Next-Generation Sequencing (NGS) identified an EGFR L858R mutation (VAF 80%). First-line Osimertinib induced a dramatic clinical and radiological response within days. However, disease progression occurred after 9 months. A repeat biopsy and NGS re-evaluation revealed a persistent L858R mutation with an increased VAF (89%), without secondary resistance mutations (T790M, C797S) or bypass alterations, suggesting non-genetic resistance mechanisms.

**Conclusion:**

This case underscores the critical difficulty of diagnosing lepidic-patterned tumors in an oncological emergency. It highlights the necessity of a multidisciplinary approach, combining cytology, radiology, and early molecular testing as a surrogate for traditional histopathology to guide urgent targeted therapy.

## Introduction

Lung cancer is one of the most common cancers worldwide with high incidence and mortality, heterogeneous clinical features, and complex histologic and molecular patterns. Non-small cell lung cancer (NSCLC) accounts for the majority of cases (approximately 85%), with adenocarcinoma being one of the most frequent subtypes [1, 2]. Bronchoalveolar carcinoma (BAC) is a rare subtype of NSCLC that accounting for 3%–6% of lung cancer, usually occurs in the lung periphery along alveolar walls, without invasion of the lung parenchyma [3]. Since 2015, World Health Organization (WHO) has replaced the term BAC from the 2010 WHO classification with “lepidic adenocarcinoma,” based on the predominant histopathological pattern where lepidic growth is the most prominent feature. The lepidic growth pattern involves the proliferation of atypical pneumocytes or Clara cells along the alveolar walls [4]. Besides, lepidic adenocarcinoma often coexists with other invasive components, requiring the pathologist to carefully assess the percentage of each component. Minimally Invasive Adenocarcinoma (MIA) is a key concept related to the lepidic subtype, which is defined as a solitary adenocarcinoma ≤3 cm in diameter with a predominant lepidic pattern and an invasion size of ≤5 mm. This feature confers an excellent prognosis, with a nearly 100% 5-year survival rate after complete surgical resection [4, 5]. Meanwhile, the invasive adenocarcinoma with a predominant lepidic pattern is the group of tumors with an invasive component greater than 5 mm where the lepidic pattern is predominant. Although the prognosis is not as favorable as MIA, it is still better than other subtypes such as solid or micropapillary [4, 5]. Identifying the predominant pattern is critical as it is strongly associated with patient prognosis. However, the accurate diagnosis of this subtype in clinical practice presents numerous challenges [6–8]. Even though morphology remains the standard principle of lung tumor diagnosis, immunohistochemistry (IHC) and molecular testing applications, including next-generation sequencing (NGS) in small biopsies or cytology specimens, are extremely useful helping to determine the diagnosis. The dramatic change in advanced NSCLC management and the increasing use of minimally invasive tissue acquisition methods may be useful in the treatment of atypical phenotype lung cancer [9]. However, this case report explores the difficulties in diagnosing lepidic adenocarcinoma from histopathological criteria and other diagnostic techniques, and seeks potential solutions to improve the pathological diagnostic accuracy.

## Case Presentation

**FIGURE 1:**
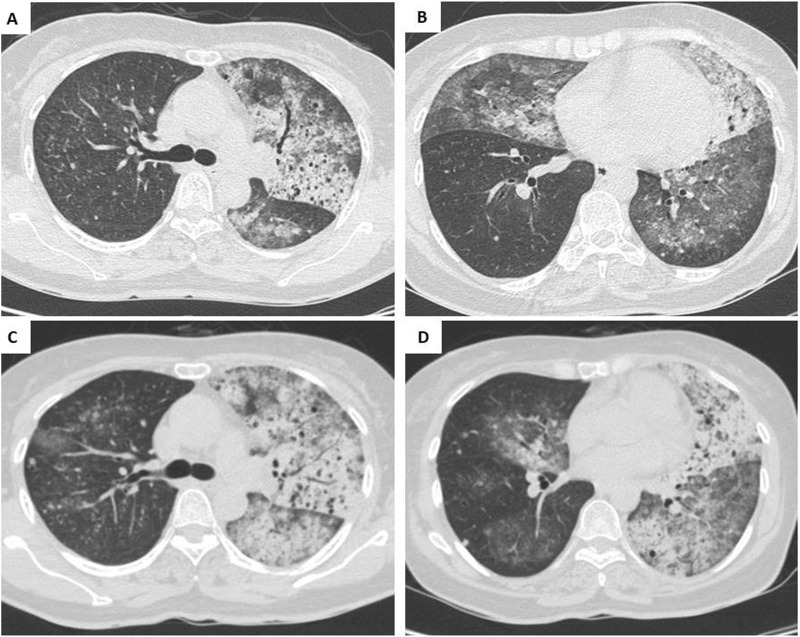
Chest CT findings prior to Osimertinib therapy. **(A,B)** Baseline scan showing air bronchograms, bilateral scattered ground-glass opacities, and multiple nodular lesions. **(C,D)** Repeat CT after 1 month showing marked progression of diffuse pulmonary lesions.

A 57-year-old, non-smoker, female patient presented with a persistent cough and sputum expectoration lasting for 3 months. Her medical history was unremarkable with no significant environmental or occupational exposure to carcinogens, and there was no known family history of lung cancer or other hereditary malignancies. Written informed consent was obtained from the patient for the publication of this case report and the associated clinical and genomic data. On 27th July 2023, she was admitted with a high volume of sputum production being recorded (>500 mL per day). On admission, the tuberculosis check-ups involving acid-fast *bacillus* (AFB) (2 samples), bronchoalveolar lavage, sputum gene Xpert, and Interferon Gamma Release Assay (IGRA) showed no abnormalities. The bronchoscopy also did not find any suspected lesion. On the chest computed tomography (CT), there was an alveolar condensation syndrome with an air bronchogram, scattered ground-glass opacities, and multiple nodular lesions in the left lung and the lower lobe of the right lung (shown in Figures 1A,B). However, the patient’s symptoms persisted despite antibiotic treatment. On 11 August 2023, she was referred for suspected AFB-negative pulmonary tuberculosis and received a 10-day anti-tuberculosis regimen, but the disease progressed, necessitating oxygen therapy at 3L/minute. By August 30th, she developed fever and bilateral crackles, with laboratory results showing a high white blood cell count (30 × 10^3^/µL) and a CT scan confirming increased bilateral pulmonary lesions (shown in Figures 1C,D).

Within 2 days, her condition worsened to acute respiratory failure, characterized by tachypnea (30 breaths/min), grade 4 dyspnea, SpO_2_ dropping to 75%–85%, and requiring escalation of oxygen support to 10–15 L/min via continuous positive airway pressure (CPAP).

**FIGURE 2:**
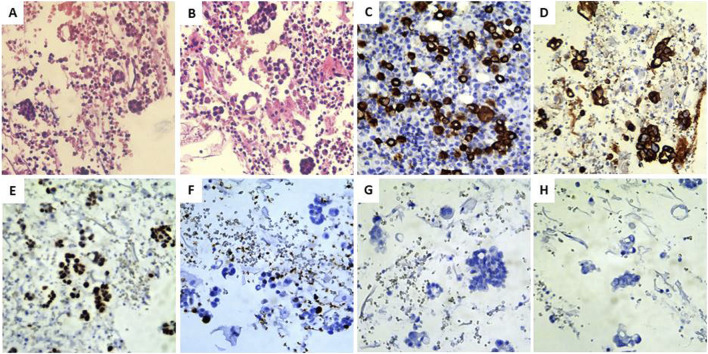
Cytological and IHC panel. **(A,B)** Sputum cell block demonstrating malignant cells. **(C–F)** Positive expression of CKAE1/3, CK7, TTF1, and Ki67. **(G,H)** Negative staining for CK20 and WT1.

After a multidisciplinary board meeting, lepidic adenocarcinoma was a hypothesis in this case. Thus, the cytologic analysis of the sputum was performed but did not show any significant findings in the sputum. The cell block revealed abnormal cells, suspicious for malignancy (shown in Figures 2A,B). These cells were oval to round and, in some areas, arranged in a linear, lepidic-like configuration that mimicked alveolar walls. Morphologically, the cells exhibited features of malignancy, including enlarged nuclei with irregular contours, conspicuous nucleoli, and abundant, vacuolated cytoplasm. To further characterize the tumor, immunohistochemical staining was performed. The tumor cells demonstrated positive immunoreactivity for CKAE1/3, CK7, and TTF1, with a positive Ki67 proliferation index (Figures 2C–F, respectively). Conversely, stains for CK20 and WT1 were negative (Figures 2G,H). Based on these cyto-morphological and immunohistochemical findings, a diagnosis of lung adenocarcinoma was confirmed. Molecular analysis was performed using NGS on the Illumina MiniSeq system (Illumina, USA). The assay utilized reagents from New England BioLabs (USA) and was designed to detect single nucleotide variants (SNVs), small insertions, and deletions (indels) within the coding regions and adjacent intronic boundaries (−20/+10 nucleotides) of a 15-gene panel. This panel included EGFR, ALK, BRAF, FGFR2, HER2, KEAP1, KRAS, MET, NRAS, NTRK, PIK3CA, RET, ROS1, STK11, and TP53. The analysis identified an EGFR L858R mutation with a high variant allele frequency (VAF) of 80.0%, while other clinically significant mutations, including EGFR T790M, C797S, exon 19 deletions, and exon 20 insertions, were not detected. Given the clinical presentation, the identified EGFR mutation was interpreted as a somatic event rather than a germline variant. Genetic counseling and further germline testing were not performed.

**FIGURE 3:**
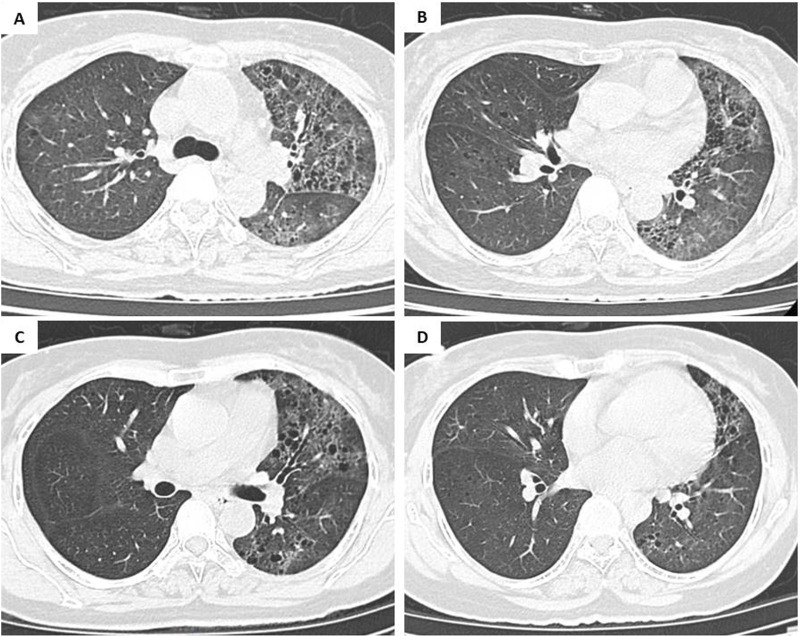
Radiologic response during follow-up. **(A,B)** Chest CT at 3 months displaying significant reduction in ground-glass opacities and nodular lesions. **(C,D)** Follow-up scan at 6 months confirming stable disease with no evidence of progression.

**FIGURE 4:**
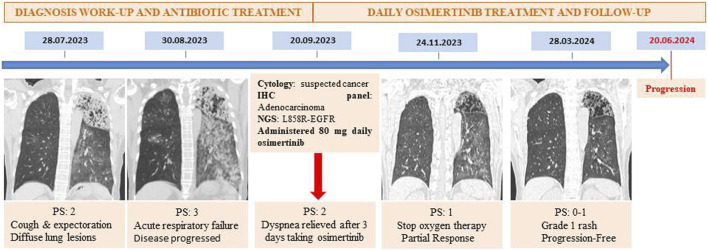
Timeline of the diagnostic work-up and therapeutic management.

The patient started treatment with 80 mg of osimertinib daily from September 20th and had a significant clinical response. After just 3 days, her SpO2 level rose to 92%–95%, allowing for the discontinuation of CPAP. Oxygen therapy was subsequently tapered, and the patient was discharged after 10 weeks of hospitalization. In the following 2 months, the patient became fully independent of oxygen and had an performance status of 0–1 according to Eastern Cooperative Oncology Group (ECOG) [10]. The CT scan in November 2023 showed that the pulmonary lesions in both lungs had significantly decreased (shown in Figures 3A,B). After 6 months of treatment, the CT scan in March 2024 continued to show a reduction in lesions with no significant lesion in the right lung (shown in Figures 3C,D). Progression-free survival (PFS) on osimertinib reached 9 months by June 20, 2024. Following disease progression, a repeat biopsy of the primary lung lesion was performed. Molecular re-evaluation using the same NGS panel confirmed the persistence of the EGFR L858R mutation with a high VAF of 89.0%. No secondary resistance mutations, such as EGFR T790M or C797S, nor any bypass signaling alterations (e.g., MET amplification or KRAS mutations), were detected within the scope of the 15-gene analysis. Figure 4 details the clinical course, illustrating the temporal relationship between the diagnostic procedures, molecular profiling, and the patient’s subsequent response to therapy.

## Discussion

The present case report highlights the lepidic adenocarcinoma diagnostic and therapeutic challenges. According to the 2021 WHO classification, a diagnosis of lepidic adenocarcinoma (formerly non-mucinous bronchioloalveolar carcinoma) requires a comprehensive histopathological evaluation of the entire tumor to accurately measure and exclude an invasive component of more than 5 mm, which distinguishes it from minimally invasive adenocarcinoma (MIA) or adenocarcinoma *in situ* (AIS) [5]. The primary problem arises from small biopsy samples themselves. Most lung cancer case are diagnosed via the biopsy methods such as transthoracic core needle biopsy, bronchoscopic biopsy, or liquid biopsy. These specimens are often very small, with not many cancer cells, presenting a major obstacle to comprehensively evaluating the tumor’s growth pattern. Due to tumor heterogeneity, a small biopsy may only sample the lepidic component while missing other invasive patterns, leading to a risk of under-diagnosing the true malignancy [5]. Furthermore, an invasive focus of ≤5 mm is hardly identified on a small needle biopsy [4, 5]. This was a significant issue in our case, where diffuse lesions made targeted biopsy difficult and delayed the diagnosis. Adding another layer of complexity is the morphological similarity between lepidic adenocarcinoma and other lesions. The lepidic growth pattern is similar to Atypical Adenomatous Hyperplasia (AAH), a pre-cancerous lesion, and Adenocarcinoma *in situ* (AIS), a non-invasive cancer [5, 11]. Differentiating these lesions is crucial as their prognosis and management are entirely different. While IHC is vital for distinguishing adenocarcinoma from other lung cancer types using markers like TTF-1, its role in differentiating between adenocarcinoma subtypes is quite limited [4]. Currently, there are no specific IHC markers for the lepidic pattern, and the diagnosis still relies predominantly on morphology on Hematoxylin and Eosin (H&E) slides [5].

In the context of an oncological emergency, a clinical-radiological diagnosis is not only pragmatic but essential. The initial presentation mimicked infectious processes such as pneumonia or tuberculosis, given the patient’s clinical symptoms and radiological findings of alveolar condensation syndrome and nodular lesions on thoracic CT imaging. In radiologic findings, reticular opacities, traction bronchiectasis, and honeycombing images can be found. In previous cases with lepidic adenocarcinoma, prolonged cough and expectoration were common symptoms of admission [7, 8, 12]. The characteristics in CT images can vary with air bronchograms, ground-glass nodules, focal fibrosis, pneumonia-like consolidation, or infectious cavitation [7, 8, 12]. The mechanism was suspected to be bronchial gland cells' hypersecretion, which also relates to EGFR over-expression and abnormal transport of transepithelial chloride and water [12]. Bronchoscopy and lung biopsy are required to differentiate BAC from other lung infections or alveolar hemorrhage. Due to diffuse bilateral pulmonary lesions without a targeted tumor, the bronchoscopy failed at the beginning of her hospitalization. The delay in diagnosis and rapid disease progression causing poor clinical conditions prevented us from performing a transbronchial biopsy or fine needle aspirate to clarify the malignancy suspicion. A similar circumstance was also reported by Asil Daoud et al [7]. If an MIA is misdiagnosed as a more aggressive invasive cancer, the patient might undergo more extensive surgery and unnecessary adjuvant therapies [5, 13]. If an invasive cancer is mistaken for a benign lesion, treatment may be delayed. This emphasizes the urgency of diagnosis in lepidic adenocarcinoma, where acute respiratory failure is a common and rapidly progressing complication. Elise Nguyen et al reported a 26-year-old female with a chronic cough, progressive dyspnea, and imaging of perihilar opacities, consolidation, and ground glass pulmonary nodule infiltrates in both lungs. After her death on the 3^rd^ day of hospitalization, autopsy and IHC revealed well-differentiated, mucinous, lepidic predominant adenocarcinoma [8]. Another case by Asil Daoud et al was a 55-year-old male patient diagnosed with diffuse pneumonic type adenocarcinoma, who passed away due to respiratory distress syndrome (ARDS) appearing shortly after admission [7].

Fortunately, the gene mutation of EGFR at L858R by NGS facilitated targeted therapy with osimertinib, resulting in a remarkable clinical response and improvement in the patient’s respiratory status within days of initiating treatment. This rapid clinical improvement underscores the importance of molecular testing in guiding targeted therapy decisions in advanced lung adenocarcinoma, particularly in cases with atypical clinical presentations or treatment resistance. The clinical management of this case was further complicated by the emergence of drug resistance after a 9-month period of progression-free survival (PFS). According to Jin Tong et al, this 3^rd^-generation TKI relieved dyspnea in a BAC patient with respiratory failure within a week and achieved a durable response for up to 16 months [12]. A repeat tissue biopsy of the primary lung lesion followed by a comprehensive 15-gene NGS panel provided critical insights into the molecular landscape of resistance. Despite the clinical progression, the EGFR L858R mutation remained persistent with a remarkably high VAF of 89.0%. Notably, the analysis was negative for common secondary gatekeeper mutations, such as EGFR T790M or C797S, and bypass signaling alterations including MET amplification or KRAS mutations within the tested panel. This high VAF suggests that the tumor remained heavily dependent on the EGFR signaling pathway. However, the lack of typical genetic resistance markers suggests the involvement of non-genetic mechanisms, such as therapy-induced senescence [14]. In this state, dormant cells act as a critical reservoir for relapse, where recurrent escape from senescence promotes genomic instability and chromosomal transitions. This evolutionary bottleneck allows for the emergence of resistant populations characterized by high copy-number alteration burden and loss of heterozygosity, rather than the acquisition of tertiary EGFR mutations or MET amplification [14]. Furthermore, resistance can be driven by the activation of alternative survival signaling, such as the STAT3 or AKT pathways, or the suppression of pro-apoptotic proteins like Bim [15]. These complex, large-scale genomic structural changes and proteomic rewiring often fall outside the detection limits of standard 15-gene panels.

Overcoming these diagnostic challenges requires close multidisciplinary collaboration among pathologists, radiologists, surgeons, and oncologists. Improving specimen quality is also critical. To improve specimen quality and diagnostic accuracy in future cases where a lepidic pattern is suspected, we recommend a multifaceted approach. First, the early integration of Rapid On-Site Evaluation (ROSE) during biopsy procedures is crucial to ensure adequate cellularity for both morphological assessment and molecular testing [16]. In addition, for patients who can tolerate bronchoscopic procedures, transbronchial cryobiopsy should be considered over conventional core needle biopsy. Cryobiopsy allows for the acquisition of significantly larger and higher-quality tissue fragments with preserved alveolar architecture. This preservation is essential for the pathologist to confidently identify lepidic growth along alveolar walls and accurately measure invasive components [17]. When physical biopsy is prohibited by the patient’s condition, the early use of high-sensitivity NGS on liquid biopsy remains the most viable surrogate to initiate life-saving targeted therapy.

## Conclusion

The diagnosis of lepidic adenocarcinoma, especially according to the updated 2021 WHO classification, is a complex and challenging task. Limitations related to small specimen size, morphological overlap with benign and pre-cancerous lesions, and the lack of specific immunohistochemical markers pose significant difficulties for pathologists. However, by enhancing multidisciplinary collaboration and improving specimen quality, we can improve diagnostic accuracy. This will not only help in determining the most appropriate prognosis and treatment plan for each patient but will also play a crucial role in improving treatment efficacy and quality of life for individuals with lung cancer.

## Data Availability

The raw data supporting the conclusions of this article will be made available by the authors, without undue reservation.
